# Prevalence and Risk Factors for Human T-Cell Lymphotropic Virus (HTLV) in Blood Donors in Brazil—A 10-Year Study (2007–2016)

**DOI:** 10.3389/fmed.2022.844265

**Published:** 2022-03-09

**Authors:** Carolina Miranda, Denise Utsch-Gonçalves, Fabiana Chagas Camargos Piassi, Paula Loureiro, Isabel Gomes, Maísa Aparecida Ribeiro, César de Almeida-Neto, Paula Blatyta, Luiz Amorim, Sheila Oliveira Garcia Mateos, Edward L. Murphy, Brian Custer, Anna Barbara F. Carneiro-Proietti, Ester C. Sabino

**Affiliations:** ^1^Programa de Pós-Graduação em Infectologia e Medicina Tropical, Faculdade de Medicina, Universidade Federal de Minas Gerais, Belo Horizonte, Brazil; ^2^Departamento de Propedêutica Complementar, Faculdade de Medicina da Universidade Federal de Minas Gerais, Belo Horizonte, Brazil; ^3^Fundação Hemope, Recife, Brazil; ^4^Department of Clinical Medicine, Universidade de Pernambuco, Recife, Brazil; ^5^Faculdade Ciências Médicas de Minas Gerais, Belo Horizonte, Brazil; ^6^Fundação Hemominas, Belo Horizonte, Brazil; ^7^Fundação Pró-Sangue, Hemocentro de São Paulo, São Paulo, Brazil; ^8^Fundação Hemorio, Hemocentro do Rio de Janeiro, Rio de Janeiro, Brazil; ^9^Department of Laboratory Medicine, Vitalant Research Institute, San Francisco, CA, United States; ^10^Department of Laboratory Medicine, University of California, San Francisco, San Francisco, CA, United States; ^11^Faculdade de Medicina da Universidade de São Paulo, Instituto de Medicina Tropical, São Paulo, Brazil

**Keywords:** HTLV-1/2, blood donors, prevalence, risk factor, sexually transmitted infections

## Abstract

It is unknown whether HTLV-1/2 prevalence has been stable or changing with time in Brazil. We present a 10-year (2007–2016) analysis of HTLV-1/2 infection in first-time blood donors from four blood banks in Brazil. The Brazilian blood centers participating in this multicenter Recipient Epidemiology and Donor Evaluation Study (REDS) are located in Recife in the Northeast and in São Paulo, Rio de Janeiro and Belo Horizonte located in the Southeast of the country. A previous REDS study using the same database from 2007 to 2009 showed that the prevalence per 100,000 donors was 222 in Recife, 83 in Belo Horizonte and 101 in São Paulo. From 2007 to 2016, HTLV-1/2 prevalence was calculated by year, blood center and birth cohort. Covariates included age, gender, schooling, self-reported skin color and type of donation. From 1,092,174 first-blood donations, in the general analysis, HTLV-1/2 infection predominated in females, donors over 50 years of age, black skin color and less educated. The average prevalence was 228 per 100,000 donors in Recife, 222 in Rio de Janeiro, 104 in Belo Horizonte and 103 in São Paulo. In the 10-year analysis, HTLV-1/2 prevalence was stable, but a trend was observed toward an increase in HTLV-1/2 infection among younger people (*p* < 0.001), males (*p* = 0.049), those with white skin color (*p* < 0.001), and higher education (*p* = 0.014). Therefore, this 10-year surveillance of the infection showed stable HTLV-1/2 prevalence overall but a trend toward increased prevalence among the younger and more educated donors despite Brazilian policies to control sexually transmitted infections being in place for more than 10 years.

## Introduction

Screening for HTLV-1/2 was introduced in Brazilian blood banks in 1993. In the routine of blood banks, all candidates for blood donation undergo pre-donation screening, which includes a questionnaire with their health history and a brief physical examination followed by serological and molecular tests for infections susceptible to transmission by blood. All serological results are recorded in databases in each blood center under the recommendation of the Ministry of Health, Brazil. Ongoing monitoring of infectious diseases markers in the blood donor population places blood banks in a privileged position to observe infection trends in the population (1). Modifications in the rates of infectious diseases in donors may be an indication of changes in the general population thus enabling new strategies to attract safe donors (2, 3).

Regarding HTLV-1/2 prevalence in blood donors in Brazil, Catalan-Soares et al. conducted in the year 2000 a pioneering epidemiological survey in all Brazilian states. It showed that HTLV-1/2 had a heterogeneous geographical distribution in which there was a predominance of infection in the Northern/Northeastern regions, intermediate prevalence in the Southeast and a lower prevalence in the Southern region (4). Later, Carneiro-Proietti et al. confirmed with data from 2007 to 2009 that the prevalence rates of HTLV-1/2 differed among the regions of the country, with the highest rate in the Northeast.

The safety of the blood supply has been a concern and it was reinforced by the change in the donor profile triggered by the onset of the HIV epidemic. Since 1989, a study has been carried out in the United States of America, the Retrovirus Epidemiology Donor Study (REDS), funded by the National Heart, Lung, and Blood Institute (NHLBI) of the National Institutes of Health (NIH) (5). Since 2006, REDS has been conducted in the US and internationally in Brazil. The Brazilian transfusion safety research program consider as one of its main objectives the safety of the blood and its products in the context of HIV/AIDS, HTLV-1/2, and other infectious diseases transmitted through it. In Brazil, the REDS project was established initially in four blood centers located in different regions of the country, including Pernambuco State in the Northeastern region and São Paulo, Rio de Janeiro, and Minas Gerais States in the Southeastern region. Recently, REDS expanded to the Amazonas State in Northern region (6).

We present here the results of a 10-year collaborative study (2007–2016) of HTLV-1/2 infection screening in first-time donors, utilizing the REDS database covering four Brazilian blood centers that have been participating in the REDS project during this period.

## Materials and Methods

### Study Design

This study has an observational design; the database was set up by the REDS program in a study carried out by four large Brazilian blood centers located in state capitals Recife (Pernambuco State), Rio de Janeiro (Rio de Janeiro State), Belo Horizonte (Minas Gerais State), and São Paulo (São Paulo State). The database contains consolidated information recorded by computer systems in the participant blood banks, encompassing all steps of the donation process, from the donor registration at the time of donation to the release of the serological results.

### Participants

We studied all eligible, first-time blood donors from January 1st, 2007, to December 31, 2016 at the four participating Brazilian blood centers. To be eligible, the candidate had to be approved as a blood donor, according to the standard operating procedures of the blood centers, which comprise the clinical screening and blood collection. There were no other exclusion criteria. Donors were classified as community or replacement donors according to their declaration at registration as to whether they came to donate as a voluntary, community donor or in the name of a specific patient (to “replace” the blood he might need).

### Serological Tests

The serum samples used in the present study were tested using the immunoenzymatic assay (EIA) for HTLV-1/2 detection (Ortho HTLV-I/HTLV-II Ab-Capture ELISA test system, Raritan, New Jersey, United States of America, or Abbott Murex HTLV I + II, Dartford, United Kingdom).

The reactive samples were sent to the central laboratory of this study, in São Paulo, for the second EIA using a kit that was different from the one used in the assay in the serological screening of the blood bank where the first sample came from (BioMérieux, Vironostika I/II, Boxtel, Netherlands; Ortho, Pencoed, England or Abbott Murex, Illinois, United States of America). A positive result was defined as a serum testing reactive in two different EIA assays. The subset of reactive samples in the first EIA and non-reactive or borderline in the second assay, performed in São Paulo, were retested at the blood bank of origin using a Western blot (WB) MP Diagnostics HTLV Blot. Western blot results were considered positive when they showed reactivity to gag (p24 or p19) and env (GD21) proteins. Reactive samples for rgp46I and rgp46II were considered HTLV-1, and HTLV-2, respectively. The absence of bands characterized the WB as negative. The presence of isolated bands characterized indeterminate WB result. If the sample was positive in WB assay, the donor was considered reactive for HTLV-1/2.

### Statistical Analysis

Statistical analysis was performed using the R statistics version 4.0.5 program (R Core Team: R Foundation for Statistical Computing. Vienna, Austria, 2021). The characteristics of the HTLV-1/2-infected blood donor included year of donation, age, gender, education, self-reported skin color, type of donation. The analysis considered the comparison of these characteristics and of the prevalence among the four participant blood centers.

The prevalence of HTLV-1/2 and 95% confidence intervals (CI) was calculated for first-time donors per year. For cases in which the result of the second test was classified as indeterminate WB, the probability of a positive result was applied to impute cases as either positive or negative. We define a positive ratio as the number of positive results in the first and second tests divided by the total number of units tested in the first test. We applied this positive ratio to define the positivity of the samples that were undefined by the WB. The prevalence was calculated as the number of positive HTLV1/2 result after the imputation divided by the number of donors, multiplied by 100,000. The comparison of prevalence between the years 2007 and 2016 was performed using the comparison of proportions test. To assess trends in the prevalence over the years, the univariate Poisson model was used. The frequency was considered to estimate the prevalence and, to analyze the birth cohort effect, the donors were stratified into five birth cohort groups: (1) born before 1980, (2) born between 1980 and 1984, (3) born between 1985 and 1989, (4) born between 1990 and 1994, and (5) born after 1995.

The association between qualitative variates was evaluated using the Chi-Square test. The level of significance considered was 5%.

### Ethical Aspects

The study protocols were approved by the National Research Ethics Commission of the Ministry of Health in Brazil (Number: 13236), as part of the International REDS Program, and by the local ethical committees in each blood center. In addition, it was also approved by the Institutional Review Board (IRB) of the University of California San Francisco, USA (Number: IRB# H5866-28606 and IRB# 11-05195).

## Results

From 2007 to 2016, a total of 1,092,174 first-time blood donations were received in the four participating centers. São Paulo represents the largest and more populated Brazilian capital. Therefore, the majority of the donations came from São Paulo, representing 36.5% of analyzed donations. Table 1 shows the donations included in the study by year and by blood center.

**Table 1:** Number of first blood donations that were collected from January 2007 to December 2016, by year of collection, birth cohort, and blood center location.

**Characteristics**	**Total**	**Number of donations N [% (CI)]**				***P*-value**
		**Recife *n* (%)**	**Belo Horizonte *n* (%)**	**São Paulo *n* (%)**	Rio de Janeiro^*^ *n* (%)	
**Year**						<0.001
2007	57,461	11,511 (20.0)	22,609 (39.3)	23,341 (40.6)	0 (0)	
2008	94,783	29,975 (31.6)	22,705 (24.0)	42,103 (44.4)	0 (0)	
2009	96,170	31,953 (33.2)	23,683 (24.6)	40,534 (42.1)	0 (0)	
2010	98,985	32,277 (32.6)	24,361 (24.6)	42,347 (42.8)	0 (0)	
2011	97,935	33,186 (33.9)	23,916 (24.4)	40,833 (41.7)	0 (0)	
2012	129,958	33,307 (25.6)	22,682 (17.5)	43,841 (33.7)	30,127 (23.2)	
2013	125,431	33,901 (27.0)	21,856 (17.4)	41,209 (32.9)	28,465 (32.9)	
2014	128,236	35,829 (27.9)	23,986 (18.7)	41,364 (32.3)	27,057 (21.1)	
2015	131,982	35,027 (26.5)	25,296 (19.2)	43,792 (33.2)	27,867 (21.1)	
2016	131,233	37,505 (28.6)	25,592 (19.5)	39,776 (30.3)	28,360 (21.6)	
	1,092,174	314,471 (28.8)	236,686 (21.7)	399,141 (36.5)	141,876 (13.0)	
**Birth Cohort**						<0.001
<1980	378,876	109,634 (28.9)	7,378 (19.4)	148,459 (39.2)	47,105 (12.4)	
1980–1984	197,959	52,939 (26.7)	46,232 (23.4)	76,034 38.4)	22,754 (11.5)	
1985–1989	248,128	68,806 (27.7)	59,424 (23.9)	92,756 (37.4)	27,142 (10.9)	
1990–1994	194,388	60,392 (31.1)	42,904 (22.1)	60,058 (30.9)	31,034 (16.0)	
1995–1999	63,002	20,926 (33.2)	12,931 (20.5)	17,006 (27.0)	12,139 (19.3)	
	1,082,353^**^	312,697 (28.9)	235,169 (21.7)	394,313 (36.4)	140,174 (13.0)	

*P-value refers to Chi-Square test*.

* *Rio de Janeiro was included in REDS program in 2012. So, donations included the period 2012–2016*.

** *There were 9,821 records missing the birth age*.

**Figure 1:**
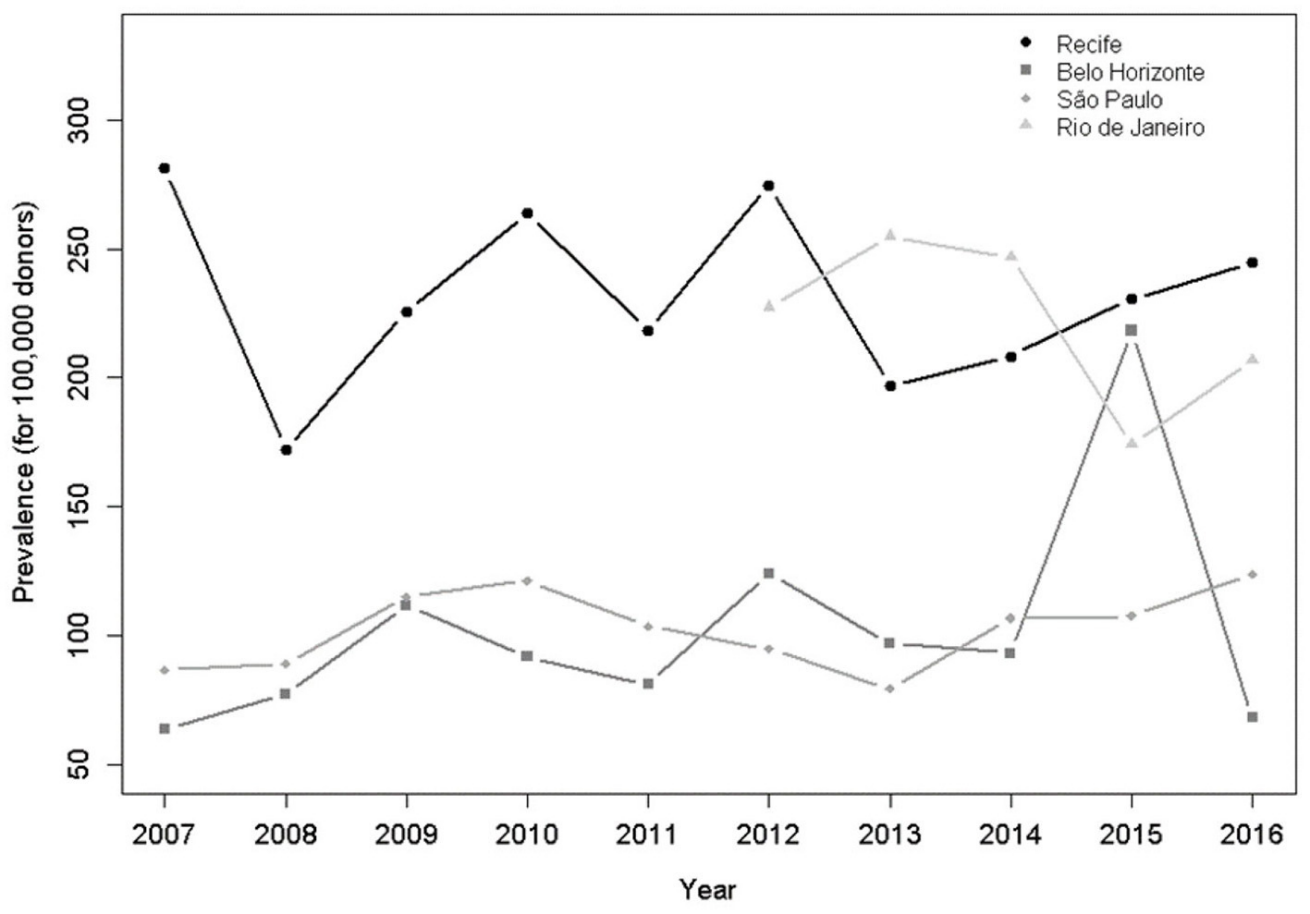
HTLV-1/2 prevalence in first-time donors per 100,000 donors per year and by blood center (2007–2016). Poisson model. Rio de Janeiro entered into the REDS study in 2012.

Figure 1 shows the prevalence of HTLV-1/2 by year and blood center over the 10-year period. HTLV-1/2 average prevalence [per 100,000 donors (95%CI)] was higher in Recife [228.3 (211.5; 245.1)] and Rio de Janeiro [222.0 (197.4; 246.6)], in relation to Belo Horizonte [103.7 (90.6; 116.8)] and São Paulo [103.3 (93.3; 113.4)]. In 2015, a peak of HTLV-1/2 infection was detected in first time blood donors in Belo Horizonte.

**Figure 2:**
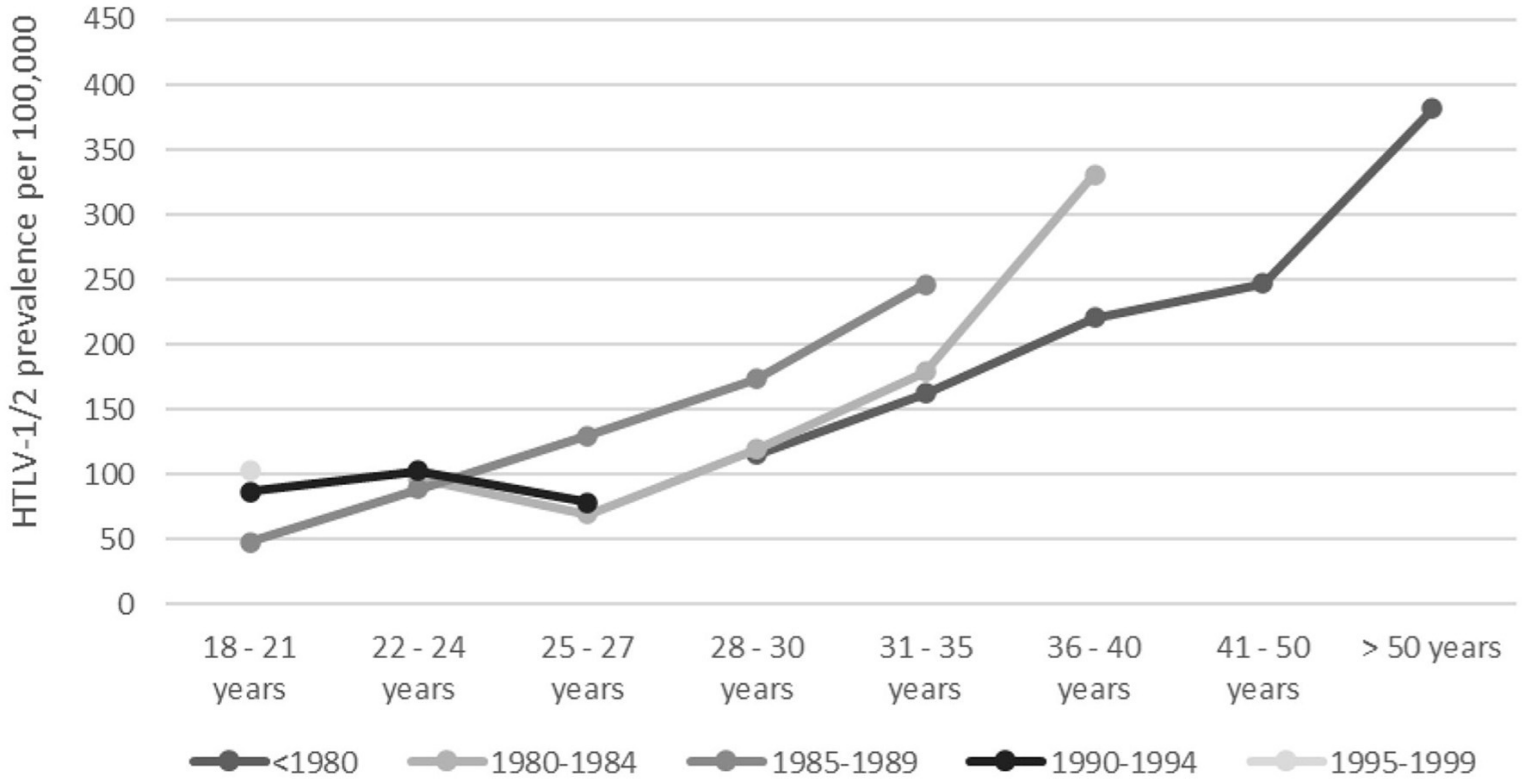
HTLV-1/2 prevalence by age and birth cohort. Dots and lines of differing color indicate birth cohorts as specified under the figure.

Figure 2 shows the effect of the birth cohort in relation to the prevalence of HTLV-1 and the aging led to an increase in the prevalence for each birth group (*p* < 0.001; Table 1).

In order to disclose variations and possible trends over the period regarding the HTLV-1/2 prevalence in relation to gender, age, schooling, skin color, and type of donation, we presented the 10-year data and compared the HTLV-1/2 prevalence of 2007 and 2016 regarding the covariates (Table 2).

**Table 2:** Comparative analysis of HTLV-1/2 prevalence (per 100,000) among first-time blood donors in the years 2007 and 2016 regarding gender, age groups, schooling, skin color, and type of donation.

**Characteristics *N* per 100,000**	**Period**			**Variation**	***P*-value**
	**All 10 years**	**2007**	**2016**	**(%)**	
**Blood center**					
Recife	228.3 (211.5; 245.1)	281.5	244.6	−13.1	0.563
Rio de Janeiro^*^	222.0 (197.4; 246.6)	227.3	206.7	−9.1	1.000
Belo Horizonte	103.7 (90.6; 116.8)	63.8	67.9	6.4	0.218
São Paulo	103.3 (93.3; 113.4)	86.5	123.8	43.1	0.658
**Gender**					
Female	179.6 (167.5; 191.7)	128.5	169.9	32.2	0.207
Male	136.0 (136.7; 145.3)	108.7	161.9	48.9	**0.046**
**Age (years)**					
<20	86.0 (71.7; 100.3)	44.4	88.0	98.2	0.303
21–29	103.9 (94.3; 113.6)	57.4	112.1	95.3	**0.032**
31–39	184.5 (168.4; 200.6)	161.6	209.8	29.8	0.349
41–49	244.5 (218.2; 270.9)	259.2	243.6	−6.0	0.943
50+	361.4 (315.9; 407.0)	322.0	354.2	10.0	0.934
**Schooling**					
Elementary	240.1 (219.6; 260.7)	116.7	253.7	117.4	**0.001**
Middle	138.4 (128.5; 148.4)	76.2	174.4	128.9	**0.003**
College	90.5 (78.3; 102.6)	45.8	89.4	95.2	0.369
**Skin color**					
Black	206.1 (179.6; 232.5)	95.3	221.9	132.8	**0.045**
White	109.0 (99.1; 119.0)	78.7	129.3	64.3	0.063
Mixed	174.1 (161.7; 186.4)	98.7	177.9	80.2	0.035
Other	154.9 (85.3; 224.5)	267.7	147.8	−44.8	0.936
**Donor type**					
Community	140.8 (130.8; 150.7)	107.8	137.9	27.9	0.298
Replacement	171.2 (159.8; 182.6)	118.8	199.5	67.9	**0.009**

* *The year 2012 was the reference*.

**Figure 3:**
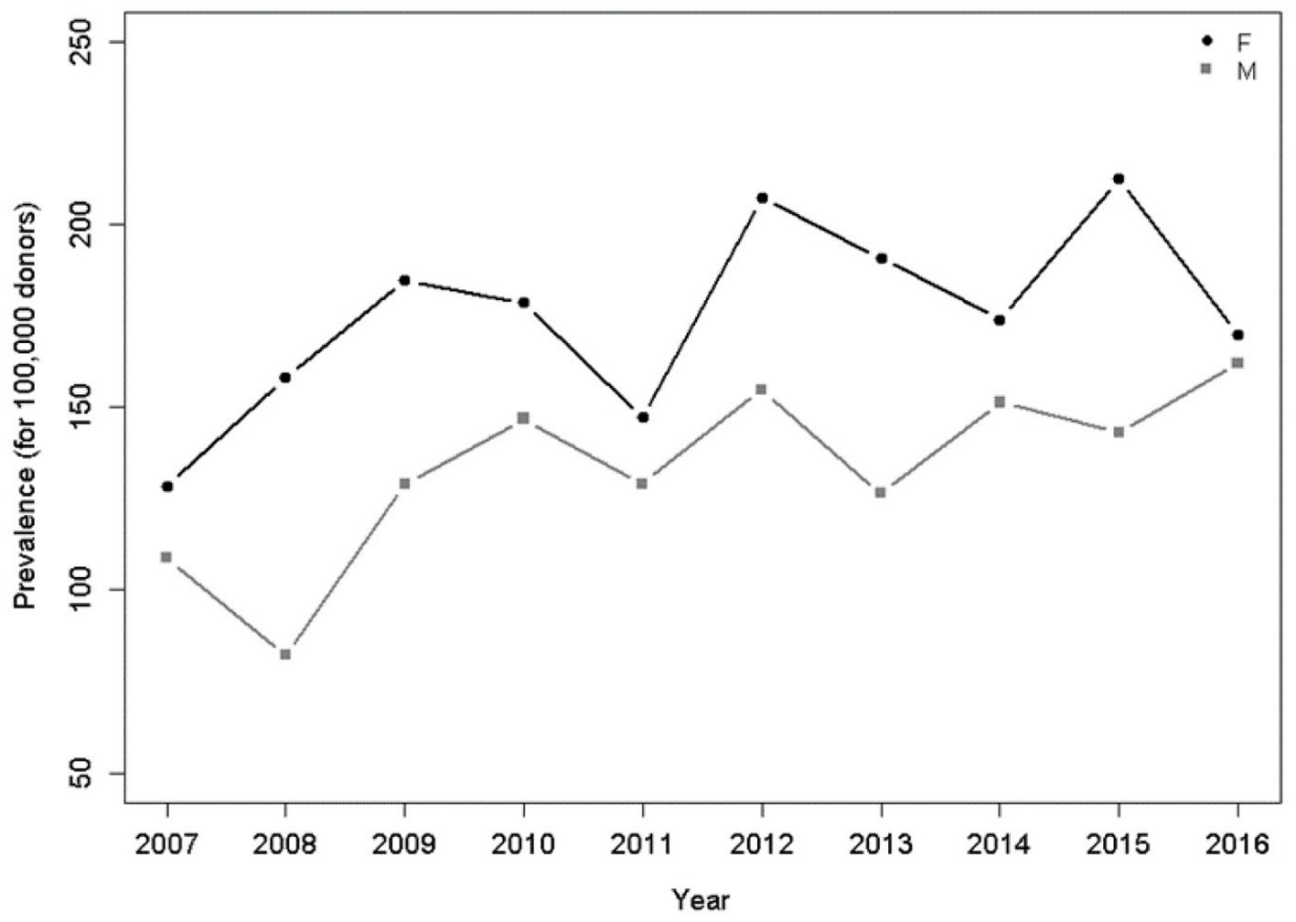
Prevalence per 100,000 donors per year and gender. There was a significant increasing trend for men (*p* = 0.049), Poisson model.

The analysis by gender showed that HTLV-1/2 prevalence was higher among women (Table 2), although there was a longitudinal progressive increase among men (Figure 3).

**Figure 4:**
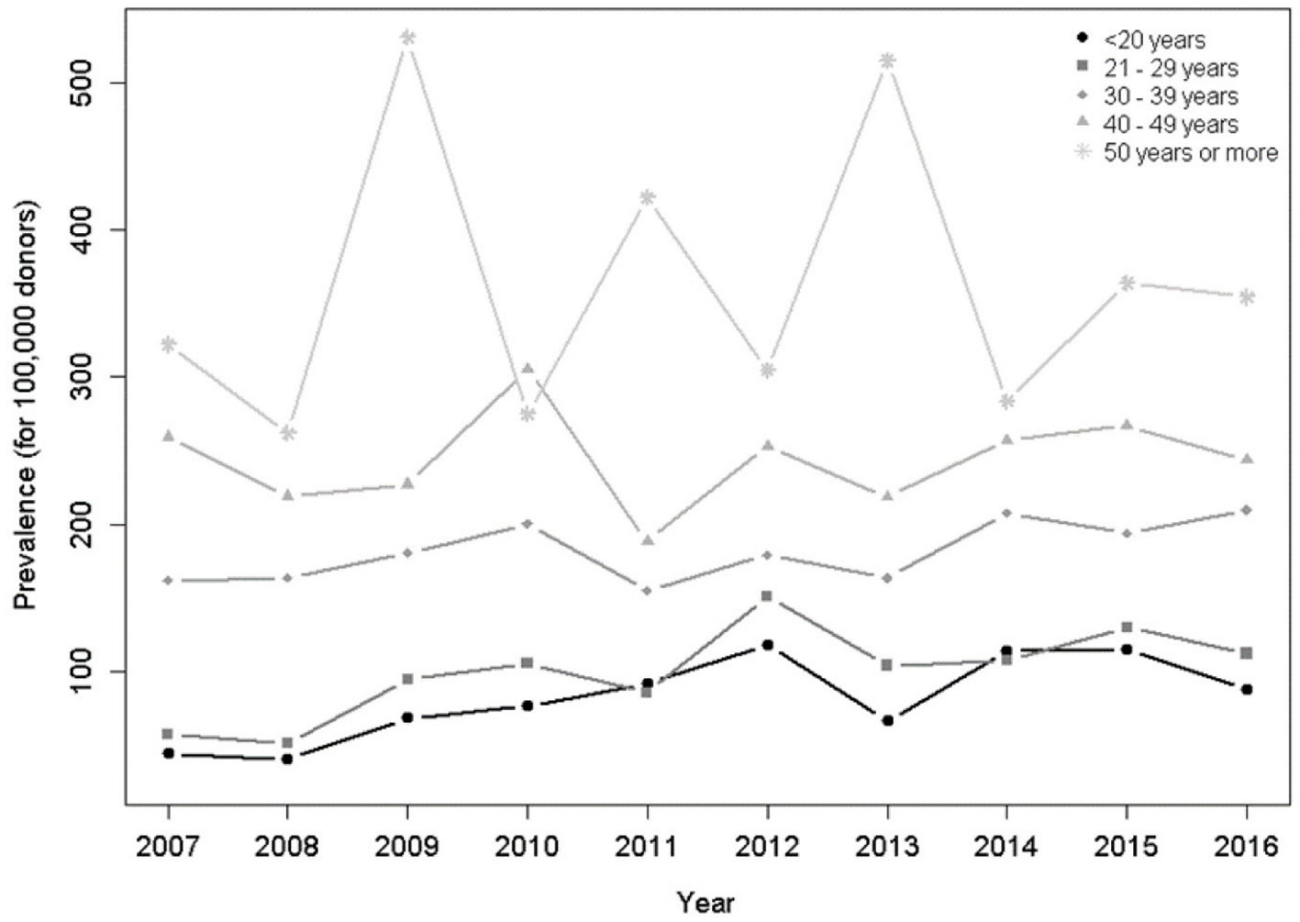
Prevalence per 100,000 donors per year and age group. There was a significant increasing trend for groups <20 years (*p* = 0.014), 20–29 years (*p* < 0.001), in the Poisson model.

In relation to age groups, during the 10-year period, there was a trend toward an increase in the seropositivity for individuals under 20 years of age (*p* = 0.014) and between 20 and 29 years of age (Figure 4).

**Figure 5:**
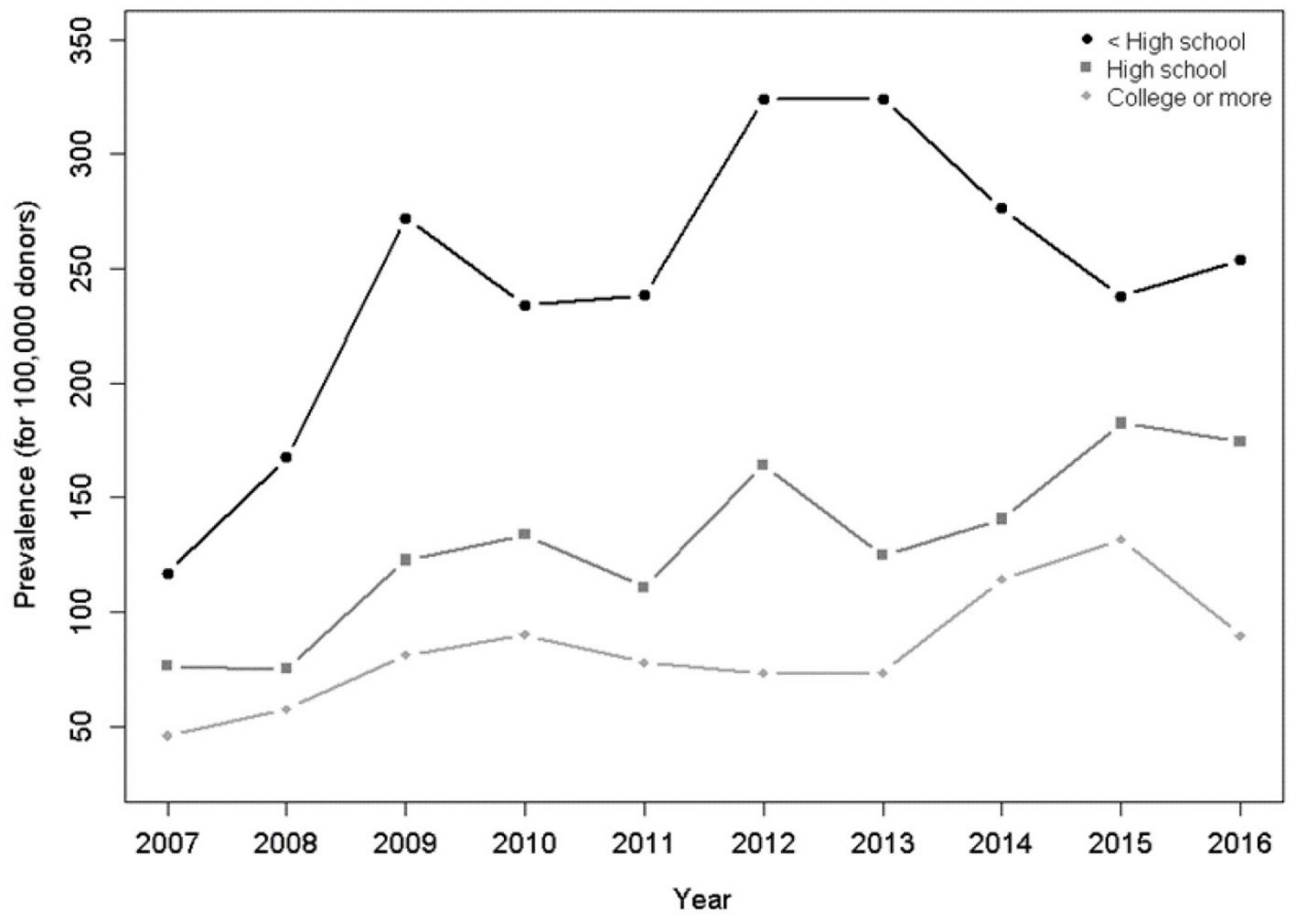
Prevalence per 100,000 donors per year and education level. There was a significant increasing trend for HTLV-1/2-infected donors who studied less than high school (*p* < 0.001), up to high school (*p* < 0.001), and higher or more (*p* = 0.014), in the Poisson model.

Regarding educational attainment, the 10-years analysis showed an increasing trend in the HTLV-1/2 seropositivity for the lowest level (*p* < 0.001) and for the highest level of education (Figure 5).

**Figure 6:**
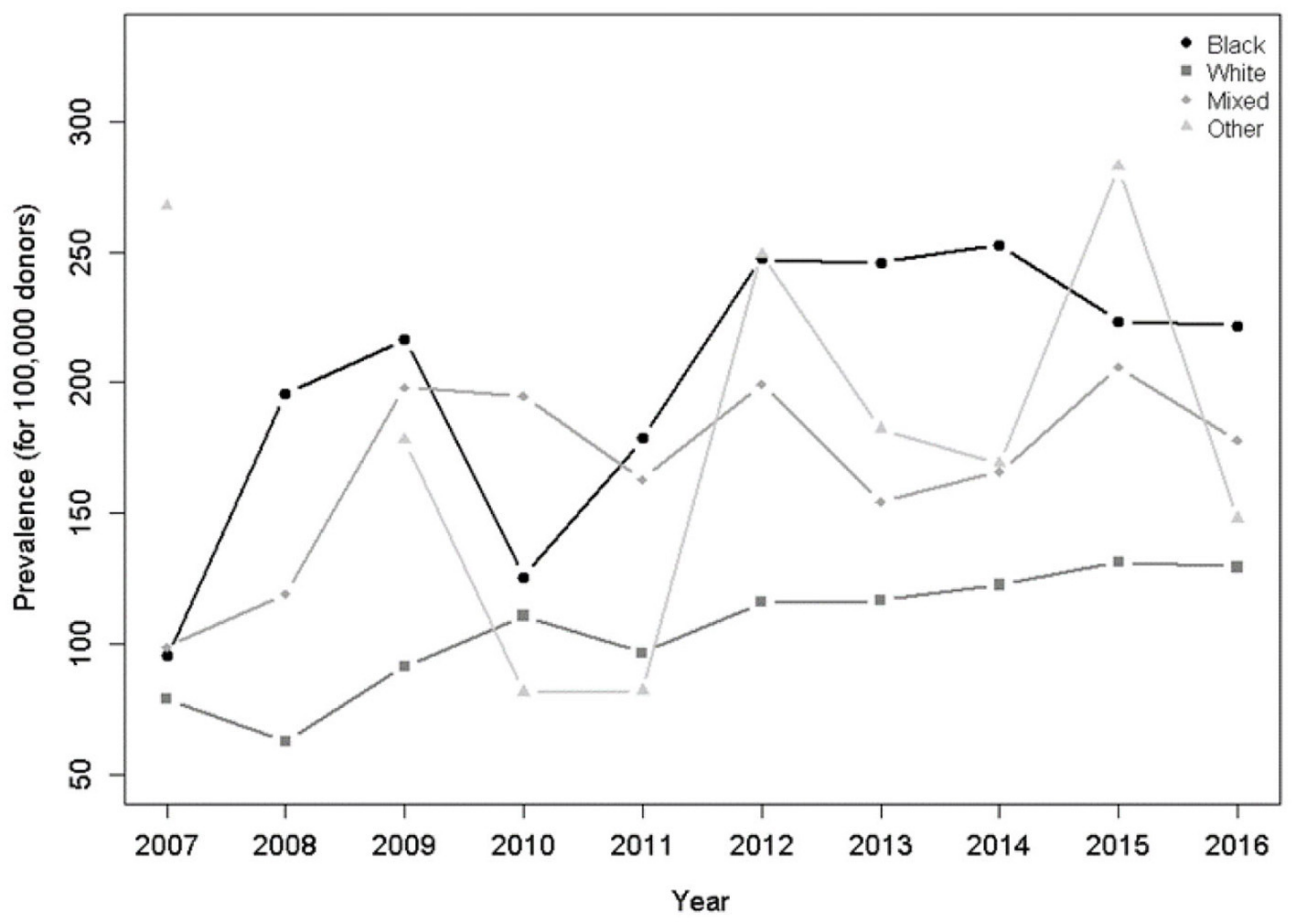
Prevalence per 100,000 donors per year and skin color. There was a significant increasing trend for blacks (*p* = 0.023) and whites (*p* < 0.001) in the Poisson model.

Regarding the self-declared skin color, black skin color predominated in the general analysis (*p* = 0.045; Table 2) and a stronger trend in the HTLV-1/2 seropositivity was observed in people with white skin color (*p* < 0.001) compared to black skin color (*p* = 0.023) during the 10-year period (Figure 6).

**Figure 7:**
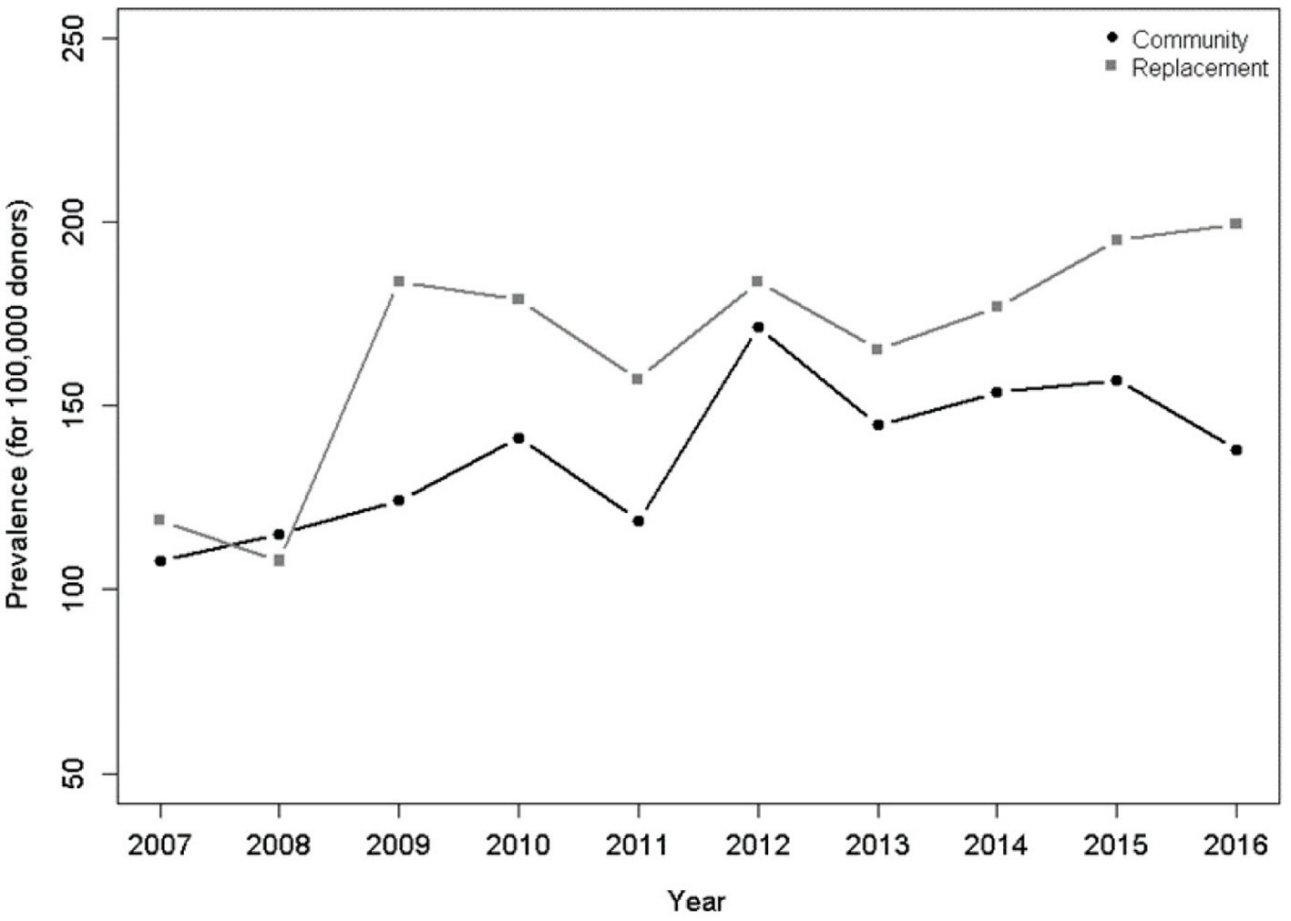
Prevalence per 100,000 donors per year and type of donor. There was a significant upward trend for both groups, including community donors (*p* = 0.025) and donors of replacement (*p* < 0.001) in the Poisson model.

Regarding the type of donation, replacement donors had higher HTLV-1/2-seroprevalence when compared to community donors (Table 2). Both types of donors showed a trend of increased seropositivity over the years (Figure 7).

In Table 3, we presented the HTLV1/2 prevalence by blood center and covariates. Females were more frequent in Belo Horizonte (60.6%) and São Paulo (61.5%) when compared to Recife (59.3%) and Rio de Janeiro (56.3%). Infected individuals with upper education were more frequent in São Paulo and Rio de Janeiro (< 0.001). In relation to skin color, the prevalence of people who self-reported black skin and mixed skin was higher in Recife in comparison to Rio de Janeiro and São Paulo (< 0.001). Regarding the type of donation, the rates of community donors vs. donors of replacement varied, and Rio de Janeiro and São Paulo presented the larger number of infected individuals who were community donors, which means individuals who come spontaneously to donate. Supplementary Table 1 shows the prevalence of all the covariates per year.

**Table 3:** HTLV-1/2- prevalence (95% confidence interval) from 2007 to 2016 (10 years) in the four participating blood banks.

**Characteristics**	**Recife**	**Belo Horizonte**	**São Paulo**	**Rio de Janeiro**
**Gender**				
Female	287.5 (255.2; 319.8)	125.9^*^ (105.4; 146.5)	127.8^*^ (111.7; 143.9)	255.6 (215.0; 296.2)
Male	197.6 (178.4; 216.9)	82.0^*^ (65.8; 98.4)	80.0^*^ (67.7; 92.3)	198.6 (167.9; 229.2)
**Age (years)**				
≤ 20	116.2 (87.8; 144.7)	77.8 (49.5; 106.1)	51.6^*^ (31.8; 71.5)	106.1 (59.6; 152.6)
20–29	152.1 (129.9; 174.3)	79.6^*^ (62.4; 96.8)	65.2^*^ (52.6; 77.9)	159.8 (125.2; 194.3)
30–39	282.7 (245.7; 319.8)	113.0^*^ (85.1; 140.9)	118.7^*^ (97.5; 139.8)	260.3 (208.8; 311.7)
40–49	351.1 (292.9; 409.4)	146.5 ^*^ (98.7; 194.3)	185.9^*^ (148.8; 223.1)	307.8 (229.4; 386.2)
≥50	605.2 (487.0; 723.5)	289.7^*^ (186.2; 393.2)	211.2^*^ (157.4; 265.1)	438.1 (319.3; 556.9)
**Schooling**				
Elementary	342.8 (300.0; 385.7)	142.8^*^ (114.2; 171.3)	176.1^*^ (143.1; 209.1)	424.0 (327.0; 521.1)
Middle	190.2 (167.3; 213.0)	93.4^*^ (75.9; 110.9)	93.9^*^ (80.8; 107.0)	246.1^*^ (209.0; 283.1)
College	103.9 (70.9; 136.9)	74.5 (49.9; 99.2)	70.5 (53.6; 87.3)	128.1 (98.1; 158.1)
**Skin color**				
Black	306.0 (241.0; 370.9)	120.2^*^ (84.7; 155.7)	134.7^*^ (94.0; 175.4)	358.4 (268.6; 448.2)
Mixed	245.1 (219.9; 270.2)	113.0^*^ (93.6; 132.4)	124.4^*^ (104.8; 143.9)	230.2 (187.6; 272.7)
White	140.5 (112.8; 168.1)	86.5^*^ (65.5; 107.5)	78.9^*^ (66.7; 91.2)	184.9^*^ (153.9; 216.0)
Other	176.2 (0.0; 420.2)	50.5 (0.0; 149.5)	182.3 (90.1; 274.5)	216.0 (0.0; 515.0)
**Donor type**				
Community	208.4 (181.0; 235.8)	103.5^*^ (80.3; 126.8)	104.8^*^ (93.0; 116.6)	218.3 (185.4; 251.2)
Replacement	239.7 (218.5; 261.0)	103.9^*^ (87.3; 120.5)	99.4^*^ (80.3; 118.5)	234.0 (194.1; 273.8)

*P-values refers to Poisson model*.

* *Indicate the prevalence with significative difference compared to Recife prevalence (reference). P-value was omitted because it would be a P-value for each comparison of each center with the reference (Recife)*.

## Discussion

The analysis of a 10-year HTLV-1/2 surveillance in four blood centers in Brazil representing the Northeastern and Southeastern regions, which are the most populous in the country, showed that overall HTLV-1/2 prevalence has been stable from 2007 to 2016. However, during the period, there was an increase in HTLV-1/2 seropositivity among male, white skin color, more educated people and younger aged. The same trend was detected in the four blood centers (Table 3). This occurred despite blood bank policies in place since 1993 to safeguard blood transfusions from HTLV-1/2 infection as well as the public health policies aiming at the reduction of sexually transmitted infections (STIs). In Brazil, there are about 800,000 people infected by HTLV-1/2 and, certainly, the country represents one of the largest endemic areas worldwide (7, 8).

When we compared our results encompassing data from 2007 to 2016 with those published by REDS with data from 2007 to 2009 regarding the same blood banks and using the same diagnostic algorithm presented here, it is evident that the prevalence barely changed (1). This previous REDS study showed that the prevalence per 100,000 donors was 222 in Recife, 83 in Belo Horizonte and 101 in São Paulo (1). However, after the first decade of HTLV-1/2 screening in blood banks, a reduction in the prevalence of HTLV-1/2 occurred when comparing the REDS results with data from 1995 to 2000 in the Brazilian national seroprevalence study in blood centers, but done without the use of confirmatory testing and based on an EIA (4). Based on data of all the Brazilian States and the Federal District, the prevalence per 100,000 blood donors was 750 in Recife, 470 in Rio de Janeiro, 660 in Belo Horizonte, and 320 in São Paulo (4). Although the comparison of the prevalence presented in these two studies has been hampered by divergent methods of testing, there was a reduction in rates in all capitals, as expected (1, 4). The implementation of public health policies to control blood-borne diseases certainly influenced this decline.

HTLV-1/2 prevalence has been falling in other HTLV-1/2-endemic countries. A study carried out from 2000 to 2006 with data from blood donors in Nagasaki, an endemic area for HTLV-1/2 in Japan, demonstrated a reduction in the prevalence of HTLV-1/2 among people born after 1987 and the persistence of high prevalence among those born before 1960. The reasons for this declining trend in the prevalence of HTLV-1/2 include social changes over the years, such as decreased duration of breastfeeding, increased frequency of artificial feeding, migrations and common use of condoms by younger people in endemic areas (9). However, in the last two decades, the estimated number of HTLV-1 carriers has not dropped much (10).

Regarding gender, the overall prevalence in females was higher, which is a feature of HTLV-1/2 infection already known in Brazil and worldwide (1, 7, 11). The transmission has been considered to be more effective in the male-female direction during sexual intercourse (12, 13). The analysis of the 10-year period showed an increasing trend in male prevalence, which must be analyzed considering that if, on the one hand, candidates for blood donation are predominantly men, on the other hand, this preponderance has always been the same in the blood banks in an epidemiological context of higher prevalence of HTLV-1/2 in female blood donors. Thus, there seems to be occurring a change in the profile of the individual infected with HTLV-1/2 (*p* = 0.049; Figure 3).

The distribution of donors by age group was similar in the four capitals and the HTLV-1/2 prevalence increased with age, which is another known epidemiological feature of HTLV-1/2 infection and previously attributed to a birth cohort effect (1, 11, 14). We confirmed in the birth cohort analysis that the prevalence of HTLV-1/2 increased with the aging of the population (Figure 2).

The age group analysis during the 10-years period showed a trend toward an increase in prevalence of people under the age of 29, which suggests recent transmission by sex (Figure 4). Injection drug use is a possibility for increasing HTLV-1/2 but probably unlikely in the blood donor population. The recent recrudescence of syphilis and an increase in HIV prevalence among young people in Brazil corroborate the hypothesis of sexual transmission (15, 16). A study carried out in São Paulo found that a large percentage of adolescents has not learned about safe sexual behavior (17). Another study comprising 1,208 young people interviewed in the survey “Youth, Behavior and STD/AIDS” showed that 4 out of 10 Brazilian citizens aged between 18 and 29 years admitted that they did not use condom in their last sexual intercourse (18). Undoubtedly, unsafe sex among young people is an important risk factor for ISTs, which have been increasing in this age group in the last two decades in Brazil. In fact, despite social dissemination of information about sex education, it seems that the target population has not yet been effectively reached. Similarly, in Japan, despite preventive measures, the rate of infection in young men has been increasing in non-endemic areas comparing to endemic, which suggest that horizontal transmission among adolescents and adults has been happening (10).

Educational status varied among the centers, with São Paulo and Rio de Janeiro presenting a higher prevalence of HTLV-1/2-positive donors with more years of schooling (Table 3). This reflects the better economic conditions of these two capitals, which influences the access to education. It is noteworthy that São Paulo had the largest increase in HTLV-1/2 prevalence (43.1%) comparing 2007 and 2016 (Table 2). The other capitals presented a minimal increase in HTLV-1/2 prevalence (Belo Horizonte) or a decrease (Recife and Rio de Janeiro). Prospective blood donors in São Paulo have been shown to withhold information in the pre-donation interview in order to have access to free testing for sexually transmitted infections (19). The 10-years analysis about education (Figure 5) showed an ascendant line in HTLV-1 prevalence for either low or high schooling, which may indicate that educational policies in Brazil have not led to changes in sexual behavior.

Socio-economical vulnerability has largely been correlated with HTLV-1/2 infection (20–24), something which was confirmed in the present study. Several publications have reported that the indicators of more vulnerable socioeconomic markers, such as less formal education and black skin color, are associated with increased HTLV-1/2 prevalence in endemic and non-endemic areas (20, 24, 25). In addition, the higher HTLV-1/2 prevalence among self-declared black people found in the present study has already been reported and it is possibly due to the African origin of HTLV-1/2 and its dissemination to Brazil via slave trade (1).

Regarding the type of donor, we found a higher prevalence of replacement donors when compared to community donors among the HTLV-1/2-positive individuals. Donors of replacement have been reported to have a higher prevalence of several viral markers, including HTLV-1/2 infection (26).

Our study has limitations. We confirm the higher prevalence of HTLV-1/2 in Recife and Rio de Janeiro compared to São Paulo and Belo Horizonte (1), although statistics for Rio de Janeiro were only available for 2012–2016 because Rio de Janeiro entered the REDS program in 2012. Regarding the prevalence during the period, Belo Horizonte presented an isolated peak of infection in 2015 (Figure 1), which can be explained by a change of reagent lots in this blood center that occurred during the months of August and September coinciding with the peak in prevalence. We suspect that the corresponding increase in HTLV-1/2 seropositivity was due to either increased sensitivity or reduced specificity of the new assay. The circumstance that gives support to this inference is that the changing in the lots resulted in the normalization of the current prevalent value. The lack of HTLV-1/2 confirmatory testing is another limitation, although we agree that a dual EIA testing strategy is an acceptable alternative for an epidemiological study.

In conclusion, STIs have been considered a serious public health problem in Brazil due to their widespread occurrence, lack of symptoms and concurrent transmission of HIV. We believe that HTLV-1/2 infection deserves more attention within STIs prevention efforts since the current profile of the infected population includes younger people, indicating an increase in recent infection.

## Data Availability Statement

The datasets for this study can be found in dropbox.com [https://www.dropbox.com/sh/1dg5gx8s3snrb2d/AAB-c457wuFs6s6tg5igV-tRa?dl=0].

## Ethics Statement

The studies involving human participants were reviewed and approved by National Research Ethics Commission of the Ministry of Health in Brazil number: 13236. Written informed consent for participation was not required for this study in accordance with the national legislation and the institutional requirements.

## Author Contributions

ES and BC conceived the study. ES, BC, PL, AC-P, CA-N, LA, and MR contributed to the design and implementation of the research. CM and IG processed the data and performed the analysis. IG designed the figures. DG, AC-P, EM, CM, IG, and FP contributed to the analysis of the results and to the writing of the manuscript. DG, AC-P, EM, and CM drafted the manuscript. ES, BC, PL, AC-P, CA-N, LA, MR, PB, and SG supervised the project and the data collection. All authors contributed to and approved the manuscript.

## Funding

This study was supported by the National Institutes of Health, National Heart, Lung, and Blood Institute by Grant No. HHSN268201100007I.

## Conflict of Interest

The authors declare that the research was conducted in the absence of any commercial or financial relationships that could be construed as a potential conflict of interest.

## Publisher's Note

All claims expressed in this article are solely those of the authors and do not necessarily represent those of their affiliated organizations, or those of the publisher, the editors and the reviewers. Any product that may be evaluated in this article, or claim that may be made by its manufacturer, is not guaranteed or endorsed by the publisher.
