# Supplementary material for: Prevalence and Risk Factors for Human T-Cell Lymphotropic Virus (HTLV) in Blood Donors in Brazil—A 10-Year Study (2007–2016)
Source: Front Med (Lausanne). 2022 Mar 9;9:844265. doi: 10.3389/fmed.2022.844265 (PMC8959844; doi:10.3389/fmed.2022.844265)
Supplement: Supplementary file 1 [file Table_1.pdf]

Supplementary Table 1. HTLV-1/2 prevalence per 100,000 donors (IC 95%) per year regarding gender, age, schooling, skin color and type of donation.

Characteristics	2007	2008	2009	2010	2011	2012	2013	2014	2015	2016
**Gender**										
Female	128.5 (82.5; 174.4)	158.0 (117.7; 198.3)	184.7 (142.1; 227.3)	178.5 (137.3; 219.7)	147.2 (111.0; 184.5)	207.2 (168.7; 245.7)	190.8 (153.8; 227.8)	174.0 (139.9; 208.1)	212.4 (175.9; 248.8)	169.9 (137.6; 202.2)
Male	108.7 (72.3; 144.2)	82.1 (58.4; 105.8)	129.0 (99.2; 158.8)	146.9 (115.5; 178.4)	129.0 (99.2; 158.8)	154.8 (126.6; 182.9)	126.4 (100.2; 152.6)	151.3 (122.3; 180.4)	142.9 (114.6; 171.2)	161.9 (131.4; 192.4)
**Age**										
< 20	44.4 (0.9; 87.9)	40.2 (8.0; 72.4)	68.4 (26.0; 110.8)	76.5 (31.3; 121.7)	91.9 (42.0; 141.9)	118.3 (67.7; 168.8)	66.5 (28.9; 104.2)	114.1 (66.5; 161.8)	115.6 (67.3; 163.9)	88.0 (47.4; 128.7)
21 – 29	57.4 (27.3; 87.4)	51.2 (28.8; 73.7)	94.4 (64.4; 124.4)	105.3 (73.8; 136.7)	85.7 (56.9; 114.5)	150.7 (116.9; 184.6)	104.0 (75.2; 132.8)	107.9 (78.9; 136.9)	130.1 (98.3; 162.0)	112.1 (82.3; 142.0)
31 – 39	161.6 (92.6; 230.7)	163.9 (109.7; 218.2)	180.5 (125.3; 235.7)	200.4 (143.8; 257.0)	155.1 (105.8; 204.3)	178.9 (133.7; 224.1)	163.4 (119.9; 207.0)	207.5 (158.6; 256.4)	193.9 (147.9; 239.9)	209.8 (161.1; 258.6)
41 – 49	259.2 (139.6; 378.8)	218.9 (136.4; 301.3)	226.9 (141.4; 312.4)	305.0 (208.2; 401.8)	188.2 (111.3; 265.0)	252.7 (177.3; 328.1)	218.6 (145.2; 292.1)	256.7 (176.2; 337.1)	266.8 (186.2; 347.4)	243.6 (166.2; 321.0)
50 +	322.0 (122.7; 521.2)	261.7 (129.4; 393.9)	530.4 (334.5; 726.3)	275.0 (140.4; 409.6)	421.7 (253.4; 590.1)	304.6 (187.7; 421.4)	514.7 (355.6; 673.8)	283.6 (165.2; 401.9)	363.8 (235.9; 491.6)	354.2 (227.7; 480.7)
**Schooling**										
Elementary	116.7 (75.0; 158.4)	167.5 (116.9; 218.1)	272.2 (204.5; 339.8)	233.8 (170.3; 297.2)	238.3 (173.6; 303.0)	324.4 (243.8; 384.9)	324.1 (247.2; 401.0)	276.3 (202.0; 350.6)	238.0 (167.8; 308.3)	253.7 (178.8; 328.5)
Middle	76.2 (37.7; 114.8)	75.0 (49.4; 100.6)	122.5 (91.5; 153.5)	133.6 (101.4; 165.8)	110.7 (81.7; 139.7)	164.0 (132.9; 195.0)	124.8 (97.1; 152.4)	140.5 (111.4; 169.7)	182.4 (149.8; 215.0)	174.4 (142.7; 206.1)

College+	45.8 (0; 97.6)	57.2 (17.6; 96.8)	81.0 (35.2; 126.8)	90.1 (42.9; 137.3)	77.8 (35.5; 120.1)	73.2 (42.6 103.7)	73.4 (42.7; 104.0)	114.2 (76.9; 151.5)	131.7 (94.8; 168.5)	89.4 (58.9; 119.9)
**Skin color**										
Black	95.3 (29.3; 161.4)	195.6 (109.9; 281.2)	216.8 (126.3; 307.3)	125.5 (57.3; 193.7)	178.6 (96.2; 261.1)	247.4 (164.3; 330.4)	246.0 (158.1; 334.0)	252.9 (162.5; 343.3)	223.6 (142.3; 304.9)	221.9 (139.8; 304.0)
White	78.7 (44.2; 113.2)	62.5 (37.0; 88.0)	91.3 (60.2; 122.5)	110.6 (78.9; 145.2)	96.5 (64.1; 128.9)	115.8 (86.0; 145.5)	116.6 (86.0; 147.1)	122.5 (91.5; 153.5)	131.3 (100.6; 162.0)	129.3 (98.6; 160.0)
Mixed	98.7 (50.3; 147.0)	118.9 (82.5; 155.3)	198.1 (153.9; 242.3)	194.9 (151.7; 238.1)	163.0 (124.6; 201.4)	199.4 (161.8; 237.0)	154.2 (120.4; 188.0)	165.9 (130.9; 200.9)	206.1 (167.7; 244.6)	177.9 (142.5; 213.3)
Other	267.7 (0; 638.3)	- -	178.1 (0; 424.7)	81.4 (0; 241.0)	82.0 (0; 242.8)	249.2 (0; 530.8)	182.3 (0; 434.8)	168.9 (0; 402.8)	282.8 (35.3; 530.3)	147.8 (0; 352.5)
**Type of donation**										
Community	107.8 (67.9; 147.8)	115.1 (85.2; 145.0)	124.2 (92.8; 155.6)	141.4 (107.3; 175.5)	118.7 (87.3; 150.1)	171.5 (140.1; 203.0)	144.8 (115.4; 174.2)	153.6 (123.2; 184.0)	157.1 (127.1; 187.1)	137.9 (109.9; 165.9)
Replacement	118.8 (78.9; 158.8)	107.8 (76.3; 139.2)	183.8 (144.0; 223.5)	178.9 (141.5; 216.2)	157.1 (121.8; 192.4)	183.7 (149.1; 218.4)	165.3 (131.9; 198.7)	176.8 (142.8; 210.7)	195.1 (160.1; 230.2)	199.5 (163.8; 235.2)
